# I Dare You to Punish Me—Vendettas in Games of Cooperation

**DOI:** 10.1371/journal.pone.0045093

**Published:** 2012-09-19

**Authors:** Katrin Fehl, Ralf D. Sommerfeld, Dirk Semmann, Hans-Jürgen Krambeck, Manfred Milinski

**Affiliations:** 1 Junior Research Group Evolution of Cooperation and Prosocial Behaviour, Courant Research Centre Evolution of Social Behaviour, University of Göttingen, Göttingen, Germany; 2 Department of Evolutionary Ecology, Max Planck Institute for Evolutionary Biology, Plön, Germany; Hungarian Academy of Sciences, Hungary

## Abstract

Everybody has heard of neighbours, who have been fighting over some minor topic for years. The fight goes back and forth, giving the neighbours a hard time. These kind of reciprocal punishments are known as vendettas and they are a cross-cultural phenomenon. In evolutionary biology, punishment is seen as a mechanism for maintaining cooperative behaviour. However, this notion of punishment excludes vendettas. Vendettas pose a special kind of evolutionary problem: they incur high costs on individuals, i.e. costs of punishing and costs of being punished, without any benefits. Theoretically speaking, punishment should be rare in dyadic relationships and vendettas would not evolve under natural selection. In contrast, punishment is assumed to be more efficient in group environments which then can pave the way for vendettas. Accordingly, we found that under the experimental conditions of a prisoner’s dilemma game, human participants punished only rarely and vendettas are scarce. In contrast, we found that participants retaliated frequently in the group environment of a public goods game. They even engaged in cost-intense vendettas (i.e. continuous retaliation), especially when the first punishment was unjustified or ambiguous. Here, punishment was mainly targeted at defectors in the beginning, but provocations led to mushrooming of counter-punishments. Despite the counter-punishing behaviour, participants were able to enhance cooperation levels in the public goods game. Few participants even seemed to anticipate the outbreak of costly vendettas and delayed their punishment to the last possible moment. Overall, our results highlight the importance of different social environments while studying punishment as a cooperation-enhancing mechanism.

## Introduction

Many species, especially humans, frequently cooperate and provide help to each other (for recent reviews, see e.g. [1], [2]). Cooperative behaviour prevails despite theoretical difficulties to explain its evolution. Why cooperate if one could enjoy the benefits provided by others and refrain from costly cooperative behaviour oneself? This is the so-called free-rider problem [3]. One hotly debated mechanism to prevent free-riding is *punishment* – a widespread behaviour in human interactions and in dyadic animal interactions (for reviews, see [4]–[8]). However, punishment can escalate into vendettas where “I punish you, because you punished me” leads to a vicious circle of counter-punishments. How can punishment then be beneficial for cooperation?

Punishment is understood as a behaviour that has costs for the target and somewhat lower costs to the punishing individual itself. As punishment is costly, there appears no incentive to punish. This situation is analogous to the free-rider problem of cooperation, whereby non-punishers represent second-order free-riders [9]. The second-order free-rider problem has been investigated intensively and under certain conditions punishment is evolutionary stable [10]–[15]. Moreover, an extensive amount of experimental research shows that in groups as well as dyadic interactions humans employ costly punishment and that thereby cooperation is enhanced (group games: e.g. [16]–[23]; dyadic games: [24], but see [25]). Even symbolic gestures of punishment [26] raise cooperation levels. However, earnings are usually negatively affected, because the costs of punishment cannot be compensated by higher cooperative benefits [17], [19], [23], [24]. On the other hand, if relationships last very long, negative effects of punishment costs can be overcome at the group level [21].

Previous research in the area of costly punishment has mainly concentrated on situations where punishment cannot be retaliated (e.g. [10], [18]). Under most natural conditions this is not the case. Usually punishment can be avenged by victims as has also been described in history and literature. For instance, in Shakespeare’s [27] Romeo and Juliet, the love relationship is so dramatic since the Montague and Capulet families were deeply involved in a vendetta. Vendettas are a cross-cultural phenomenon [28]. There are blood vendettas between Turkish farmers lasting as long as 60 years [29]. Vendettas occurred in the Mediterranean area in the nineteenth-century [30] and they proliferate in science [31]. Sometimes these vendettas escalate and then one reads headlines like “A 20-year feud between two neighbours […] revved up this week, ending in bloodshed” [32]. These yearlong vendettas often begin with a punishment of one party, which is perceived as unjustified by the victim [33], and turn into a continuous exchange of retributions.

Recently, there has been growing interest in the effect of retaliation on cooperative games with punishment [24], [34]–[36]. They show that humans avenge punishment regardless of its negative effect on payoffs. However, in most cases cooperation cannot be sustained by vengeful punishment. Hitherto, the possibility that punishment can escalate into vendettas has been precluded by restricting punishment to a single retaliation stage [34], [35], [37]. Though Denant-Boemont et al. [34] and Dreber et al. [24] provide the possibility for repeated counter-punishment in groups and dyads, respectively, their focus lays on another topic (neglecting the analysis of possible vendettas). In addition, Denant-Boemont et al.’s set-up provides incentives to distribute one’s punishment across all punishment rounds. Nikiforakis and Engelmann [36] study the possible outbreak of vendettas in groups where participants determined the number of punishment rounds through their behaviour. As long as (a) they punished in the previous round and (b) they had money to invest in punishment there was another opportunity to punish. Participants could invest all their remaining money in a single punishment act. Thereby a high risk of severe retaliation was generated. In this group set-up, they demonstrate that participants try to avoid vendettas by simply refraining from punishment. Vendettas occur rarely. In fact, in only about 7% of all groups was there a sufficient number of punishment rounds reached for vendettas to be possible. In a few cases, retaliatory punishment stopped because participants could no longer afford to retaliate. Hence, under these conditions the occurrence of vendettas might be reduced. Overall, the issue of whether humans (be it in a dyadic or group environment) engage in costly punishment, which can escalate into vendettas, and how these vendettas are initiated and terminated remains on the whole unanswered.

Despite the vengefulness observed in humans, theoretical research shows that punishment vendettas are not evolutionary stable. In infinitely repeated, dyadic interactions the best response to an opponent’s defection is defection and not punishment. On top of that, defection is preferred as a response to an opponent’s punishment as opposed to punishment [38]. Furthermore, a single stage of retaliation cannot stabilize groups’ cooperative behaviour in one-shot encounters [37]. Here, concealing the punisher’s identity, and thus making retaliation harder, has positive effects on cooperation. This theoretical evidence suggests that, in cooperative games, little punishment is expected and certainly no vendettas. In general, it is assumed that punishment is more likely to evolve as a mechanism to prevent free-riding in groups [8]. By contrast, punishment in dyadic interactions might be too costly – especially in light of possible vendettas – to evolve as a mechanism to maintain cooperation [8], [24], [38]. Only here, can defection be targeted specifically at free-riders without group-level detriments. Hence, if at all, only group environments provide the potential for vendettas.

In this study, we allow for vendettas by combining (a) the public goods game (PG; [39], [40]) and (b) the prisoner’s dilemma game (PD; [41], [42]) with multiple consecutive opportunities of costly punishment. This enables us to investigate our main interest: the emergence of vendettas. In line with reality, participants can thus punish their punisher in the same way immediately or later. Rational choice theory assumes that people should take this behaviour into account. Punishing social partners, who can choose to retaliate, first produces costs for punishing and second can lead to potentially higher costs when partners retaliate. This leads to exaggerated costs of punishment that should be avoided by the rational individual. In line with this, an evolutionary model [38] demonstrates that vendettas do not evolve. On the other side, however, empirical studies of costly punishment, where vendettas are impossible, show that people do indeed engage in punishment, which then stabilizes cooperation (e.g. [18]). In addition, aspects of spite (e.g. [43]) or inequity aversion [44], [45] can also motivate counter-punishment behaviour. Therefore, despite the high costs of possible vendettas, we expect participants to engage in punishment and counter-punishment in the group environment. Additionally, vendettas can be observed under natural conditions. Therefore, we expect humans also to engage in vendetta punishment under the experimental conditions of the PG. However, participants in dyadic interactions should rather abstain from punishment [8], offering no opportunities for vendettas in the PD. Hence, we will address if social interactions trigger vendettas, who starts them and who terminates them. Subsequently, we investigate how cooperative behaviour and overall payoffs in the PG and the PD will be affected.

## Methods

First-semester biology students from the Universities of Kiel, Hamburg and Münster, Germany, as well as Vienna, Austria, joined the experiment voluntarily. All participants gave their informed consent to participate. Before playing, they were informed about the scientific procedures when publishing data and that their behaviour would be collected, analysed and published without an association between participants’ real identities and behaviour in the games. Specifically, anonymity of participants was always preserved and no demographic data (e.g. gender, age) were collected during the games. By random assignment to a computer and random assignment of an alias, as well as a special payment procedure (see below) this anonymity was ensured. As it is standard in socio-economic experiments, there were no additional ethical concerns beyond that mentioned above and preserving the anonymity of participants.

For the PG, a total of 96 participants were randomly assigned into six sessions of 16 participants each. For the PD, seven sessions of six participants each were conducted (n = 42). In each session, participants were randomly seated in front of an individual computer with partitions between participants. In order not to disclose their real identity, but still allow for individual recognition within the game, participants received an alias, i.e. names of moons of our solar system (e.g. Despina or Metis), at the beginning of the games. Participants were told that they have to make decisions during the experiment whether to invest their money or not in different situations. A short introduction ensured that participants understood how to handle the computer, that they were completely anonymous throughout and after the game concerning their behaviour within the experiment, that they should not talk to one another or draw attention to them during the experiment, and that they would receive all their earnings anonymously in cash. After the experiment, each participant could collect her earnings out of an envelope entitled with her alias from behind partitions (as described in [46]). Thus, the participant herself was the only person who knew her identity in the experiment. Participants earned on average 13.41€ ±3.01 (mean ± s.d.) in the PG and 15.40€ ±6.43 in the PD.

For the PG, our experimental design follows the one of Fehr and Gächter [18] where the participants were arranged into subgroups of four individuals each, and first played a PG round followed by the possibility to punish other members of the subgroup. The starting amount was set to 20€ for each participant. In the PG situation, participants had to decide whether or not to contribute 1.00€ to a public good (this contrasts the continuous contributions in [18], as in [20], [46]). They knew that the sum of all contributions will be multiplied by 1.6 and distributed equally among all subgroup members irrespective of their contribution. In the following punishment round, participants were informed about the PG investments of all subgroup members and could then assign a punishment from 0 to 10 units for each subgroup member separately. Again, following Fehr and Gächter, each point of punishment assigned resulted in a threefold fine to be paid by the punished subgroup member. If for instance a player invested 0.30€ ( = 3 units) to punish somebody, the account of the punished member was reduced by 0.90€ ( = 9 units). Negative earnings were possible, but after the experiment ended those participants (i.e. 4 out of 96) were informed that their account balance was zero. The difference from Fehr and Gächter [18] is that instead of just one, a sequence of five punishment rounds was played after the initial PG round. In these successive punishment rounds participants knew exactly who punished whom with how much money for each subgroup member.

In the dyadic set-up, the six participants were randomly assigned into subgroups of two. Participants first played the PD which was then followed by five successive rounds of punishment. Participants received a starting amount of 20€ each, as in the PG set-up. For the PD, they had to decide whether or not to contribute 1.00€, the sum of all contributions would be multiplied by 1.6 and distributed equally among both subgroup members irrespective of contributions. Accordingly, mutual cooperation (defection) yielded a payoff of 0.60€ (0.00€), being exploited resulted in a loss of −0.20€ and a gain of 0.80€ for the one who exploited. In the following punishment rounds, participants were informed about the PD outcome and could then assign a punishment to their subgroup member. The punishment incurred a cost of 0.50€ to the punisher and a threefold fine of 1.50€ to the target (i.e. in contrast to the PG set-up where punishment was continuous). Then punishment amounts were announced to both players and the next round of punishment followed. Negative earnings occurred for 1 out of 42 participants and, as in the PG, she was informed that her account balance was 0.00€.

In each session, 16 participants in the PG played the aforementioned sequence of rounds (PG followed by five punishment rounds) three times ( = three periods). Participants in the PD played five periods, each consisted of the sequence of a PD followed by five punishment rounds. Participants were not informed about the number of PG or PD rounds nor the number of punishment rounds. Between each period, participants were reshuffled into new subgroups of four or two individuals in a way that excluded any kind of reputation building (i.e. participants received no information about the previous behaviour of new subgroup members) and direct reciprocity between periods. Being aware of this condition, no participant was able to meet a previous subgroup member in later periods again.

For statistical analyses SPSS 18.0.2 and R 2.12.1 were used. A 5%-level of significance is used and probabilities are reported as two tailed. Furthermore, for both games analyses were done on the session level, if not stated otherwise. Exceptions are the generalized linear mixed models where session effects are considered in terms of random factors.

## Results

### Cooperation in the Public Goods Game and the Prisoner’s Dilemma Game

As the research design is partially adapted from Fehr and Gächter [18], we also applied their statistical analysis where applicable. Our results show that the level of cooperation in the PG rounds increased (period 1∶47.9±15.6; period 2∶65.6±7.7; period 3∶71.9±8.6; comparing period 1 vs. period 3; Wilcoxon signed-rank test: Z = 2.21, n = 6, p<0.05). In the PD, cooperation did not change over periods (period 1∶78.4±7.8; period 2∶78.6±12.4; period 3∶80.71±6.1; period 4∶85.7±14.9; period 5∶78.6±12.4; comparing period 1 vs. period 5; Wilcoxon signed-rank test: Z = 0.38, n = 7, p = 0.71).

### Punishment after the Social Interactions

**Figure 1:**
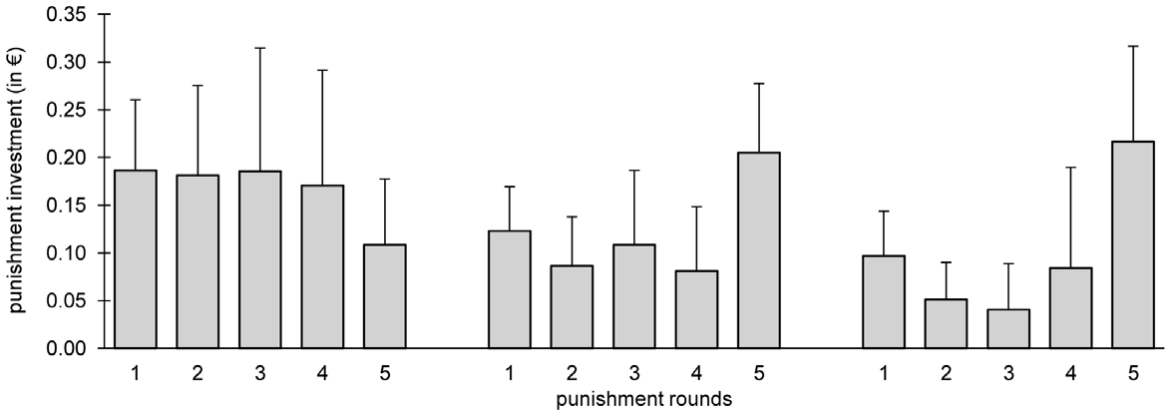
Average punishment investment (+ s.d.) per participant in the public goods game. In each of the three periods, participants played one round of public good followed by five rounds of punishment.

Punishment was frequent in the PG set-up. In overall 15 rounds of punishment and with the possibility to punish up to three subgroup members, 85.4% of the participants punished at least once; 52.1% at least five times; and 21.9% at least 10 times. Within period 1 investment in punishment did not change over the five rounds of punishment (see Fig. 1; Friedman test: χ^2^ = 2.12, df = 4, n = 6, p = 0.71). However, we found significant changes in periods 2 and 3 (period 2: χ^2^ = 11.42, df = 4, n = 6, p<0.05; period 3: χ^2^ = 14.08, df = 4, n = 6, p<0.01). In period 1, participants did not yet know the total number of rounds played in each period, afterwards they could guess. In periods 2 and 3, we observed an increase in punishment investment in the very last round. To analyse this last round effect, we compared punishment in the last and the second-last round. The respective differences were significantly different in period 2 (Wilcoxon signed-rank test: Z = 2.20, n = 6, p<0.05) and we found a trend in period 3 (Z = 1.58, n = 6, p = 0.12). Further analysis revealed that the high punishment investment in round 5 was due to few participants (in each period: 10 out of 96), who invested high amounts to punish (period 2∶0.85€ ±0.23; period 3∶0.91€ ±0.19). These participants avenged their punishment of round 4 (period 2∶30%; period 3∶26%), but also delayed their revenge of being punished in rounds 1 to 3 (period 2∶40%; period 3∶47%).

Multiple rounds of punishment in the PG allowed participants to punish back after receiving a fine. Indeed on average up to 80% of all punishing participants retaliated their punishment in a given period (period 1∶0.80±0.09; period 2∶0.58±0.20; period 3∶0.42±0.17). Within acts of punishment there was a significant relationship between punishment investment by the punisher and counter-punishment investment by the target. We used generalized linear mixed models (GLMMs), in which we included *punisher identity* and *target identity* nested within *sessions* as random factors, to model the received *counter-punishment* (0 to 10 units) in the current round as a function of *original punishment* (1 to 10 units) in the previous round. GLMMs were fitted by Laplace approximation assuming Poisson error distribution. We found that, the higher the original fine, the higher the counter-punishment (from round to round: intercept = −0.61 to −1.03, s.e. = 0.21 to 0.30, p<0.05; β = 0.15 to 0.23, s.e. = 0.04 to 0.06, p<0.05).

To analyse the motives of participants in the PG to punish we used GLMMs to model *punishment* (0 to 10 units) as a function of *participant’s and target’s PG decisions*, *subgroup members’ PG decisions*, and *provocation* (i.e. in punishment rounds 2 to 5 the punishment investment by the target in the previous round). We controlled for differences in *periods* and in *participants*, who are nested within *sessions*, and included these as random factors. We looked at the given models for each punishment round separately; hereby allowing motives for punishment to differ between rounds. GLMMs were fitted by Laplace approximation assuming Poisson error distribution. The variance inflation factors are all less than 1.25, which indicates that multicollinearity is not a problem in the models’ estimations [47]. In punishment round 1, the participant’s and her target’s behaviour in the PG predicted the punishment investment of the participant, i.e. if both contributed then punishment became less likely (see Tab. 1). In subsequent rounds of punishment the importance of the PG behaviour varies. However, the behaviour of the two other subgroup members is now important, as the more of them contributed, the more likely the punishment of the target became. In addition, the previous amount of punishment by the target significantly increased investments by the participant to punish the target in the current round.

**Table 1:** Results of the generalized linear mixed models to model punishment investment in the public goods game.

	round 1	round 2	round 3	round 4	round 5
intercept	−3.36 ***	−3.45 ***	−3.64 ***	−3.52 ***	−2.97 ***
	(0.33)	(0.43)	(0.49)	(0.38)	(0.35)
P contributed and T did not	2.55 ***	0.71 **	1.07 ***	−0.20	−0.02
contribute into the PG 1	(0.20)	(0.25)	(0.24)	(0.24)	(0.17)
P did not contribute and T	0.72 **	0.82 ***	0.83 ***	0.39	−0.47 ***
contributed into the PG 1	(0.27)	(0.22)	(0.23)	(0.23)	(0.14)
P and T did not contribute	1.33 ***	0.72 **	0.84 ***	−0.61 *	−0.17
into the PG 1	(0.26)	(0.26)	(0.24)	(0.28)	(0.18)
other two subgroup members’	0.17	0.22 *	0.28 **	0.47 ***	0.30 ***
behaviour in PG 2	(0.10)	(0.11)	(0.11)	(0.12)	(0.09)
provocation	n/a	0.48 ***	0.42 ***	0.43 ***	0.20 ***
		(0.04)	(0.03)	(0.03)	(0.02)

Provided are the estimates, the standard errors in brackets and the p-values as * p<0.05,

** p<0.01,

*** p<0.001.

The *period*, the *participant’s identity* and the *session* were added as random factors in all models (n = 864, in each round 96 participants could punish up to three subgroup members in three periods). For punishment in round 1 no previous provocation (in terms of punishment investment by the target in the previous round) is possible.

1 The contribution of both, the participant (P) and her target (T), into the public good (PG) served as reference group of the categorical fixed factor *participant’s and target’s PG decisions*.

2 The behaviour of the remaining two subgroup members was coded as 0, 1, or both contributed into the PG.

**Figure 2:**
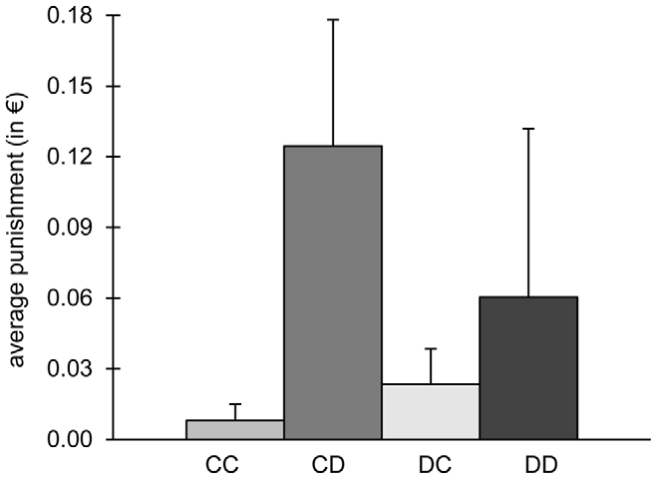
Average punishment investment (+ s.d.) in the first round of punishment in the public goods game (pooled over all periods). Participants could either contribute into the public good, C, or defect, D. Hence, in CD a contributor punished a defector (CC, DC, DD, respectively; Friedman test: χ^2^ = 12.2, df = 3, n = 6, p<0.01).

In line with the results from the GLMMs for punishment round 1, punishment was directed at non-contributing (i.e. defecting) participants. In particular, contributors, who punished defectors, spend the most money on punishment (see Fig. 2; punishment significantly differs between outcomes of PG behaviour of punisher and target: Friedman test, χ^2^ = 12.2, df = 3, n = 6, p<0.01). In all subsequent rounds the punishment investment did not differ according to the PG behaviour of the punisher and the target (see Fig. S1). This is in line with the GLMMs, as they showed that now the behaviour of other subgroup members and provocations gained importance.

In contrast to the group interactions, punishment was rare in the PD set-up (therefore, we only provide a brief analysis here). In overall 25 rounds of punishment, 30.2% of the participants punished at least once. Moreover, in every punishment round on average only 0.81±0.35 participants punished (see also Fig. S2, S3). Multiple rounds of punishment allow participants to punish back after receiving a fine. Of those few that were actually punished about 35% retaliated their punishment (period 1∶0.65±0.25; period 2∶0.59±0.26; period 3∶0.18±0.27; period 4∶0.20±0.34; period 5∶0.11±0.20).

### Vendettas of Costly Punishment

**Figure 3:**
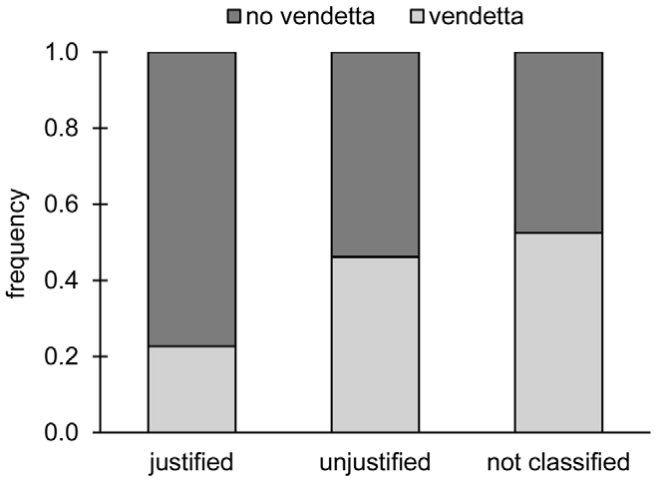
Frequencies where a participant in the public goods game punished a subgroup member in punishment round 1 and either a vendetta or no vendetta occurred (pooled over all periods). Punishment was classified as justified if a contributing participant punished a non-contributor (n = 106, individual level); it was termed unjustified if a non-contributing participant punished a contributor (n = 26); all other cases were rather ambiguous and not further classified (n = 42).

For both games, a minimum of three sequential punishments was defined as a vendetta, i.e. player A started by punishing player B, who retaliated this punishment, and was again punished by player A in the next round. In the PG set-up, vendettas were frequent. We observed 71 vendettas in total (i.e. on average 0.99 vendettas occurred in a given subgroup per period; whereas [36] observed 0.03 vendettas per subgroup). On average participants were involved in 1.48±0.88 vendettas and vendettas lasted on average 3.89±0.39 rounds (period 1∶3.72±0.24; period 2∶4.06±0.61; period 3∶3.92±0.17). Often vendettas ended because the final round of punishment was reached (46% of 71 vendettas). Otherwise, in 30% (24%) of the time a non-contributor (contributor) stopped the on-going punishment sequence. In addition, a clear pattern arises when looking at punishment in round 1 and whether a vendetta developed or not on the level of individual interactions (see Fig. 3). Justified punishment of a non-contributor by a contributor was most frequent, but did not lead to vendettas in most cases (77%). All other punishments, i.e. unjustified punishment of a contributor by a non-contributing participant and ambiguous punishment (a contributor punished a contributor; or non-contributor punished a non-contributor), triggered a vendetta in about 50% of the time. Those players engaging in vendettas pay large costs, since it includes their punishment investment and counter-punishment fines. Comparing average payoffs of participants that were involved in vendettas (10.44€ ±4.25) and participants that were never involved in a vendetta (i.e. neither started one nor did counter-punish that resulted in a vendetta; 17.05€ ±1.29) showed, that the latter earned significantly more money (sign test: n = 6, p<0.05). This is also true for players retaliating punishment (11.87€ ±2.84) versus players refraining completely from retaliation (17.15€ ±1.34; sign test: n = 6, p<0.05).

On the contrary, we observed only 12 vendettas in the PD set-up (i.e. on average 0.11 vendettas occurred in a given dyad per period). On average participants were involved in 0.57±0.69 vendettas, however, in three sessions (i.e. 45 dyads) no vendetta occurred at all. Vendettas lasted on average 3.20±0.33 rounds (period 1∶3.25±0.35; period 2∶3.00±0.00; period 3∶3.00±0.00, period 4∶3.75±1.06; period 5∶3.00). Vendettas occurred in mutual cooperative relationships (58%), when a cooperator started to punish a defector (25%), or when a defector started to punish a cooperator (17%). Vendettas never occurred in mutual defective relationships (though this was a rare outcome in general). Often vendettas ended because the final round of punishment was reached (58% of 12 vendettas). Otherwise, in 33% (8%) of the time a cooperator (defector) stopped the on-going punishment sequence.

## Discussion

Vendettas occur under natural conditions [28]–[30]. Being in a group environment, the participants in our experiment of a public goods game with five rounds of punishment opportunities frequently retaliated (i.e. immediate counter-punishment) and engaged in vendettas (on average 1.5 per participant), too. This happened even though vendettas, i.e. at least three sequential rounds of punishment, are cost-intense, as one has to pay costs for punishing and costs for being punished, multiplied by several instances. This contrasts earlier findings for groups in a different punishment environment. Here vendettas were rare [36]. Despite the costliness of vendettas and their inefficiency (i.e. they significantly reduced earnings compared to players, who abstained completely from vendettas) we observed an-eye-for-an-eye counter-punishment, where high punishment was answered with high counter-punishment. This supports the view that counter-punishment possibly escalating into vendettas is due to an attempt to restore equity between participants [33], [44], [45], [48], [49]. In the example of fighting neighbours, both see themselves as victims and both go on to restore (subjective) justice. The initial social interaction of the PG was relevant for the first punishment, i.e. defectors attracted the highest punishment. In later rounds, players primarily reacted to provocations (previous punishment). In addition, participants in the PG relied on the behaviour of other subgroup members as a social reference point: the more those cooperated the more likely the remaining subgroup member “deserved” punishment. This possibility of social comparison might explain the enhanced punishment and the more frequent outbreak of vendettas in the group environment compared to the dyadic environment. The durations of vendettas were rather long. In fact, participants’ vendettas lasted on average about four out of five rounds. Vendettas in the PG normally started with an unjustified punishment (i.e. a non-contributor punished a contributor) or when the meaning of the punishment was rather ambiguous (i.e. a contributor punished a contributor; or non-contributor punished a non-contributor). When the punished individual had defected and was “properly” punished by a cooperative participant vendettas seldom started (i.e. only in 23% of all cases; in [36] all vendettas within a period were triggered by defecting individuals). Vendettas ended out of various reasons: non-contributors (30%) or contributors (24%) stopped punishing or the final punishment round was reached (the number of rounds was not announced to participants).

Despite the occurrence of costly punishment, retaliations, and even vendettas in the group environment, the frequency of cooperation increased over time. This occurred even though direct reciprocity and reputation building between PG rounds were excluded. This result contradicts earlier findings where the vengeful response to being punished alone cannot sustain cooperation [34], [35].However, allowing for further escalations in terms of vendettas can maintain cooperation [34], [36]. The increase of cooperation in the PG is presumably due to the effect of the first punishment round where high amounts of punishment were targeted at defecting participants. Punishment of non-contributors as a direct response to their defection, excluding the possibility of vendettas, is also observed in previous studies [18], [19]. Nevertheless, in experiments earnings are usually negatively affected (e.g. [17], [24]), which is especially true for participants, who engaged in retaliation and vendettas in this experiment. In line with our findings, other experimental studies also report unjustified punishment, which in general has been termed anti-social punishment [22]. However, the evolution of cooperation is not supported in the presence of anti-social punishment [50]–[52]. In our study, anti-social punishment acts frequently led to vendettas, making the original unjustified or anti-social punishment very costly. Given that punishment can escalate, this could serve as means to reduce anti-social punishment to a minimum in long-term relationships.

Remarkably, by predicting after the first period the given PG set-up of the second and third period some participants were able to avoid costly vendettas. These participants delayed their punishment to the expected last round of punishment. This is in line with findings that participants, who were able to control the duration of possible punishment acts, in the majority tried to avoid retaliating punishment [36]. In addition, our participants invested high amounts to punish, indicating a final revenge for being punished in previous rounds where they patiently refrained from immediate counter-punishment to avoid the danger of paying counter-punishment fines themselves.

In contrast to the results of the public goods game, we find that vendettas are rare in dyadic relationships of the prisoner’s dilemma game. In three out of seven sessions, these participants did not even engage in a single vendetta. Though, vendettas are scarce different social rules seem to operate compared to the ones we observed in the group environment. In the dyadic environment, defectors did not feel the need to end an on-going punishment sequence and vendettas ceased to exist faster, by half a round. In sum, punishment and retaliation were infrequent in the PD, also because in the dyads participants acted mostly cooperative. This occurred even though we had excluded reputation building and direct reciprocity. Overall, our results are in line with the conjecture that punishment as a mechanism to maintain cooperation is conditioned on environmental attributes such as the number of interaction partners [8]. To that effect, costly punishment is more frequent in group environments to control free-riders and less efficient in dyadic environments where reciprocal defection constitutes a superior way to sanction free-riders [8], [38].

Our results for the group environment are in accordance with earlier findings that humans are willing to punish and retaliate (e.g. [6], [19], [34], [35]). We extended this line of research by showing that acts of punishment, when common in groups, can escalate into vendettas. However, the behaviour of our participants is in contradiction to theoretical postulations that vendettas should not occur under natural selection [37], as defection is the proper response evolving after provoking punishment [38]. Nevertheless, a tendency to avenge can also be found in animals [4], [53]. For instance, Japanese macaques sometimes use indirect revenge against an aggressor’s kin [54]. These counter-aggressive acts seem to have regulatory effects, as they happen in the presence of the aggressor, who however is unable to intervene. Thus these acts can serve as means to reduce the likelihood of further attacks of the aggressor against the revenging individual. Vendettas in human societies are also attributed a functional quality [30], [55]. For one, vendettas are thought to provide rules for escalating conflicts and thereby they might reduce the likelihood of full escalation (e.g. death of innocents). Additionally, social norms prescribe which kind of behaviour is to be avenged. Here, we also found that vendettas in the PG occur only under certain circumstances: after unjustified or ambiguous punishment, but rarely after justified punishment. Such counter-punishments could relate to social norms like “showing strength” or “avoiding to lose face” and are in line with biblical norms such as “an eye for an eye”. Furthermore, “natural” vendettas occur more frequently in regions where institutional punishment is rather weak or absent [55]. Considering real-world observations, we find it worthwhile to investigate multiple rounds of punishment in an experimental setting where punishment can be peer-based, but also institutionalized. Due to assured institutionalized punishment, peer-punishment might become less important, resulting in a reduced likelihood of vendettas. That cooperation is promoted by institutional punishment has been shown theoretically [56], but whether this inhibits vendettas on the peer-level remains a topic of future research. In addition, switching of partners could also avoid vendettas, especially in dyadic relationships. This preference to switch partners has been reported from reef fish who stop interacting with a cheating cleaner fish when they have access to several cleaners. However, sole access to a single cleaner leads to punishment of cheating behaviour to change the quality of the cleaner service [57]. Humans also prefer to end dyadic relationships with uncooperative partners [58] and possibly treat counter-punishing partners in a similar way. The importance of institutionalized punishment and partner switching as potential mechanisms to prevent escalations into costly vendettas has to be investigated further.

## Supporting Information

Figure S1 **Average punishment investment in the public goods game for the (a) second, (b) third, (c) fourth and (d) fifth round of punishment.**
(PDF)

Figure S2 **Average number of punishing participants in the prisoner’s dilemma game for the (a) first, (b) second, (c) third, (d) fourth and (e) fifth round of punishment.**
(PDF)

Figure S3 **Average number of punishing participants in the prisoner’s dilemma game.**
(PDF)
