# Supplementary material for: I Dare You to Punish Me—Vendettas in Games of Cooperation
Source: PLoS One. 2012 Sep 19;7(9):e45093. doi: 10.1371/journal.pone.0045093 (PMC3446949; doi:10.1371/journal.pone.0045093)
Supplement: Figure S1 — Average punishment investment in the public goods game for the (a) second, (b) third, (c) fourth and (d) fifth round of punishment. (PDF) [file pone.0045093.s001.pdf]

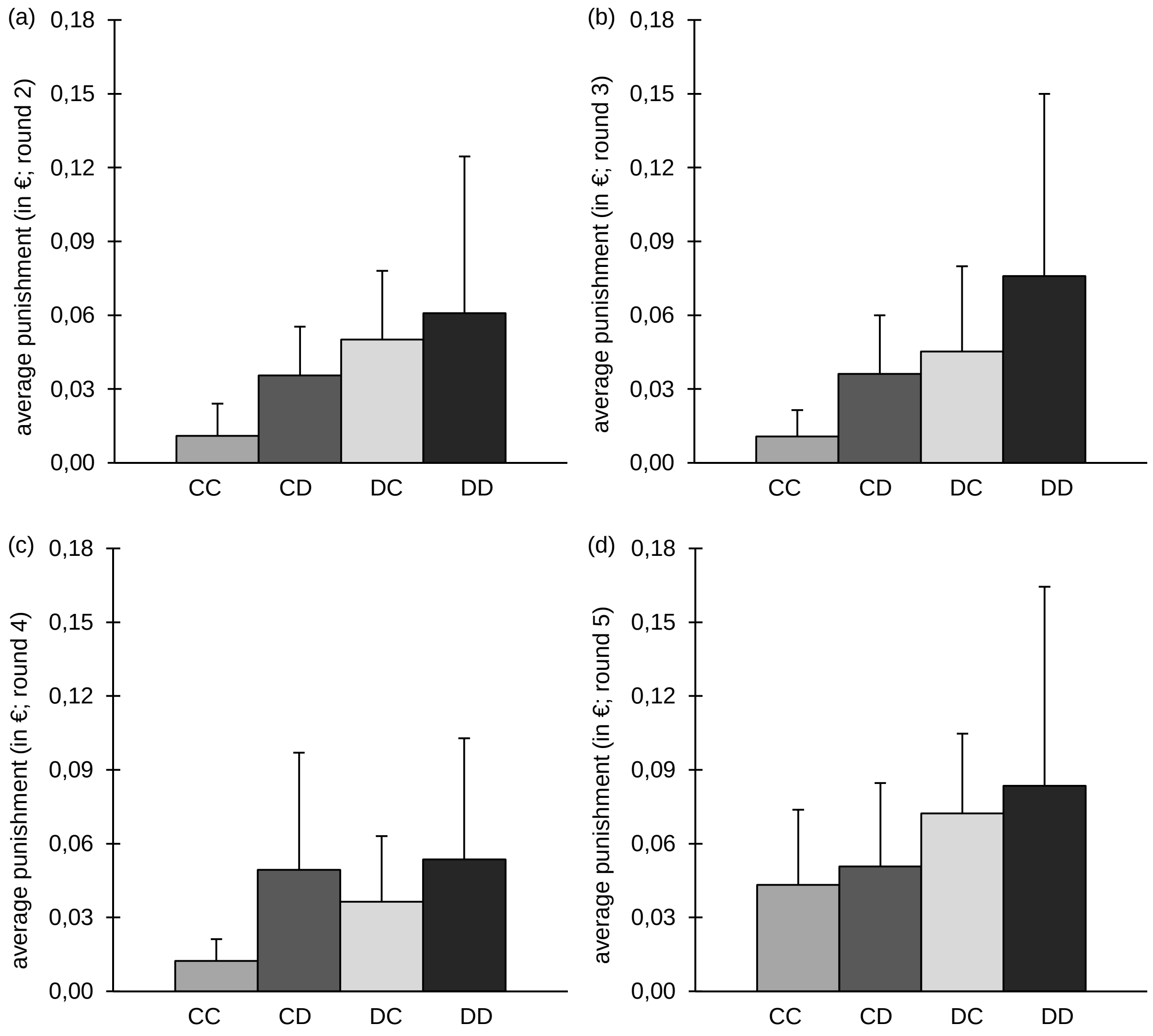

Figure S1. Average punishment investment (+ s.d.) in the public goods game for the (a) second, (b) third, (c) fourth and (d) fifth round of punishment (pooled over all periods). Participants could either contribute into the public good, C, or defect, D. Hence, in CD a contributor punished a defector (CC, DC, DD, respectively; Friedman test: (a) $\chi^2 = 5.0$, df = 3, n = 6, p = 0.17; (b) $\chi^2 = 2.95$, df = 3, n = 6, p = 0.40; (c) $\chi^2 = 2.29$, df = 3, n = 6, p = 0.52; (d) $\chi^2 = 2.6$, df = 3, n = 6, p = 0.46).
