# Supplementary material for: I Dare You to Punish Me—Vendettas in Games of Cooperation
Source: PLoS One. 2012 Sep 19;7(9):e45093. doi: 10.1371/journal.pone.0045093 (PMC3446949; doi:10.1371/journal.pone.0045093)
Supplement: Figure S2 — Average number of punishing participants in the prisoner’s dilemma game for the (a) first, (b) second, (c) third, (d) fourth and (e) fifth round of punishment. (PDF) [file pone.0045093.s002.pdf]

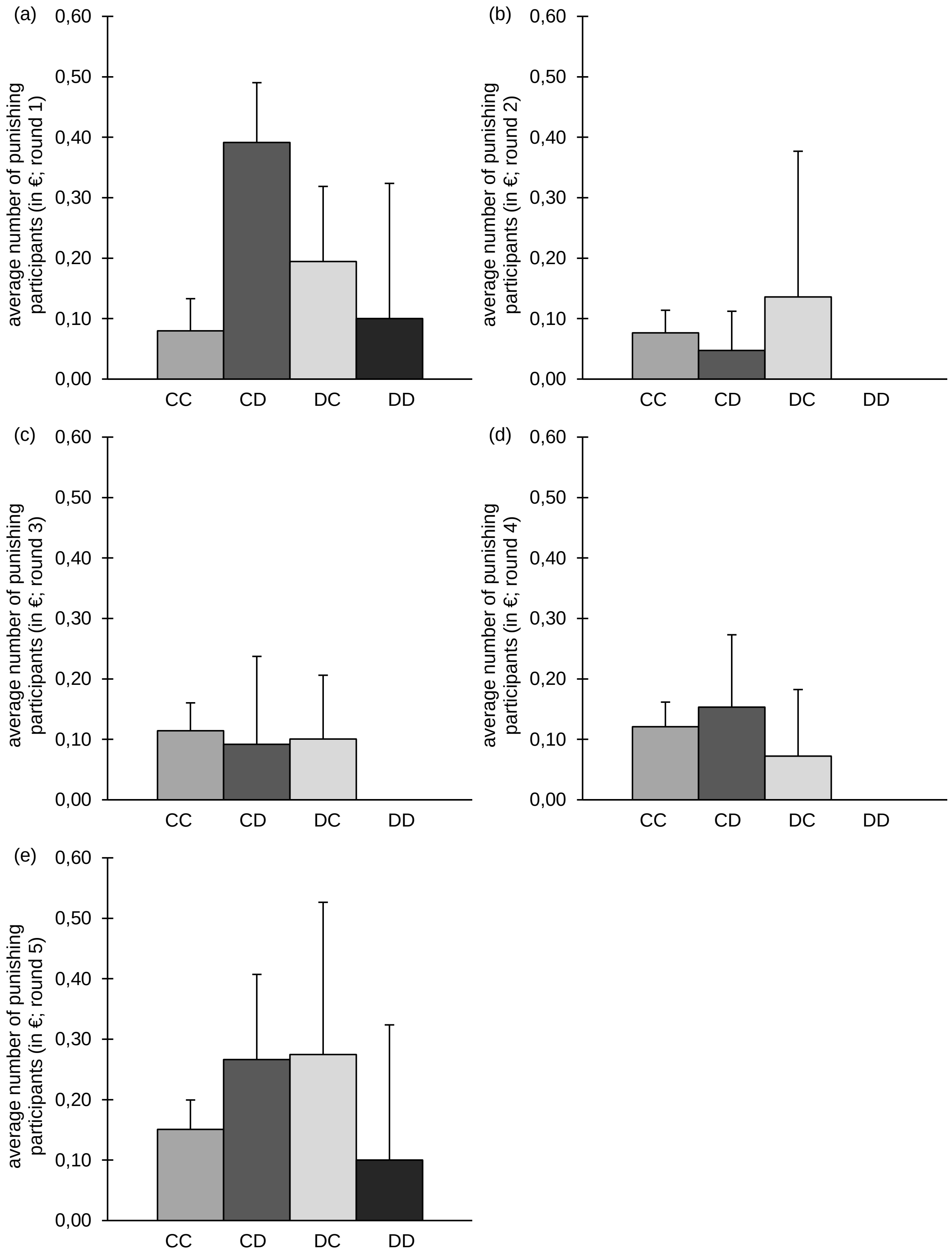

Figure S2. Average number of punishing participants (+ s.d.) in the prisoner's dilemma game for the (a) first, (b) second, (c) third, (d) fourth and (e) fifth round of punishment (pooled over all periods). Participants could either cooperate, C, or defect, D. Hence, in CD a cooperator punished a defector (CC, DC, DD, respectively).
