# Supplementary material for: I Dare You to Punish Me—Vendettas in Games of Cooperation
Source: PLoS One. 2012 Sep 19;7(9):e45093. doi: 10.1371/journal.pone.0045093 (PMC3446949; doi:10.1371/journal.pone.0045093)
Supplement: Figure S3 — Average number of punishing participants in the prisoner’s dilemma game. (PDF) [file pone.0045093.s003.pdf]

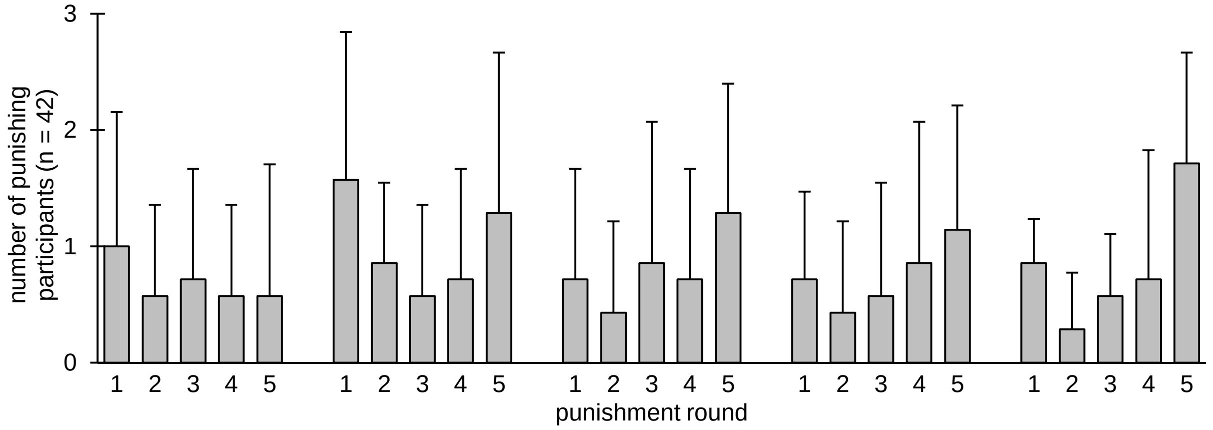

Figure S3. Average number of punishing participants (+ s.d.) in the prisoner's dilemma game. In each of the five periods participants played one round of prisoner's dilemma followed by five rounds of punishment where they could either punish or not punish.
